# Down-regulation of DcR2 sensitizes androgen-dependent prostate cancer LNCaP cells to TRAIL-induced apoptosis

**DOI:** 10.1186/1475-2867-11-42

**Published:** 2011-12-02

**Authors:** David Vindrieux, Marie Réveiller, Jacqueline Chantepie, Sadok Yakoub, Catherine Deschildre, Alain Ruffion, Marian Devonec, Mohamed Benahmed, Renée Grataroli

**Affiliations:** 1 UMR Inserm U1052/CNRS 5286, Centre Léon Bérard, 28 rue Laënnec, 69373 Lyon Cedex 08, France; 2 NYU Langone Medical Center, Department of Medicine and Pathology, 423 East 23 rd street, New York, NY 10010, USA; 3 Inserm, retired; 4 Unité de nutrition humaine, UMR INRA U1019/Université Clermont 1, Centre de recherche INRA de Clermont-Ferrand/Theix, 63122 St Genès Champanelle, France; 5 U851 Inserm-UCBL-HCL, Tour INSERM CERVI, 21 avenue Tony Garnier, 69365 Lyon, France; 6 Service d'Urologie, Centre Hospitalier Lyon Sud, 165 chemin du grand Revoyet, 69921 Oullins, France; 7 U895 Inserm, Université de Nice-Sophia Antipolis, UFR Medecine, 151 route Saint Antoine de Ginestiere, 06204 Nice, France; 8 SF Biosciences Gerland-Lyon Sud, CNRS UMS3444/Inserm US8, Université Claude Bernard Lyon 1, 50 avenue Tony Garnier, 69366 Lyon, France

**Keywords:** TRAIL, DcR2, Prostate Cancer, Apoptosis, Androgen

## Abstract

**Background:**

Dysregulation of many apoptotic related genes and androgens are critical in the development, progression, and treatment of prostate cancer. The differential sensitivity of tumour cells to TRAIL-induced apoptosis can be mediated by the modulation of surface TRAIL receptor expression related to androgen concentration. Our previous results led to the hypothesis that downregulation of TRAIL-decoy receptor DcR2 expression following androgen deprivation would leave hormone sensitive normal prostate cells vulnerable to the cell death signal generated by TRAIL via its pro-apoptotic receptors. We tested this hypothesis under pathological conditions by exploring the regulation of TRAIL-induced apoptosis related to their death and decoy receptor expression, as also to hormonal concentrations in androgen-sensitive human prostate cancer, LNCaP, cells.

**Results:**

In contrast to androgen-insensitive PC3 cells, decoy (DcR2) and death (DR5) receptor protein expression was correlated with hormone concentrations and TRAIL-induced apoptosis in LNCaP cells. Silencing of androgen-sensitive DcR2 protein expression by siRNA led to a significant increase in TRAIL-mediated apoptosis related to androgen concentration in LNCaP cells.

**Conclusions:**

The data support the hypothesis that hormone modulation of DcR2 expression regulates TRAIL-induced apoptosis in LNCaP cells, giving insight into cell death induction in apoptosis-resistant hormone-sensitive tumour cells from prostate cancer. TRAIL action and DcR2 expression modulation are potentially of clinical value in advanced tumour treatment.

## Background

Prostate cancer is the most commonly diagnosed malignancy in the male population and remains the second leading cause of cancer-related deaths in the developed world [1]. Inhibition of apoptosis is a critical pathophysiological factor that contributes to the onset and progression of prostate cancer, but the molecular mechanisms are not entirely understood. Therefore, insight into the mechanism(s) of the misregulation of apoptosis could be the basis for developing more effective therapeutic approaches to destroy apoptosis-resistant tumour cells, as found in prostate cancer [2].

Treatment with apoptosis-inducing ligands belonging to the Tumour Necrosis Factor-alpha (TNF-α) family could be an effective strategy for cancer treatment [3,4]. The best characterized ligands, Fas ligand, TNF-α, and TNF-α-related apoptosis-inducing ligand (TRAIL, also known as Apo2L) [5,6], are type II transmembrane proteins that can induce apoptosis in susceptible cells after binding to type I transmembrane receptors containing cytoplasmic "death domains". These interact with the downstream death domain-containing adapter proteins FADD or TRADD (for Fas- or TNFR-associated death domains, respectively), leading to activation of initiator caspases (e.g. caspase 8) and effector caspases (e.g. caspase 3) and apoptotic cell death [7]. Unfortunately, both TNF-α and Fas ligands have severe systemic cytotoxic effects, limiting their use as systemic agents [8]. Unlike TNF-α and FasL, TRAIL has been used effectively in systemic animal trials and has the unique feature of inducing apoptosis in cancer cells, whilst sparing normal cells [9,10]. TRAIL may therefore be a promising candidate for cancer treatment.

Transcripts of TRAIL [5,6] have been detected in many human tissues (e.g. spleen, thymus, prostate, and lung). To date, at least 4 type I transmembrane receptors have been identified, including DR4 (TRAIL-R1) [11], DR5 (TRAIL-R2) [12], DcR1 (TRAIL-R3) [13,14] and DcR2 (TRAIL-R4) [15,16]. Ligation of TRAIL with DR4 or DR5 induces trimerization of the receptor, which activates the apoptotic pathway. In contrast to DR4 and DR5, DcR1 and DcR2 act as decoy receptors for TRAIL. DcR1 is a glycosylphosphatidylinositol (GPI)-linked protein lacking an intracellular domain, and DcR2 contains a truncated death domain. They can prevent TRAIL-induced apoptosis, presumably by competing with DR4 and DR5 for binding to TRAIL [12,14].

Because they can bind TRAIL but do not signal for apoptosis, DcR1 and DcR2 appear to serve as "decoys" that inhibit apoptosis by sequestering TRAIL from the death-inducing TRAIL receptors. Moreover, Clancy et al. [17] reported that inhibition of apoptosis depends on the formation of ligand-independent complexes between DR5 and DcR2 in primary human CD8^+ ^T cells.

Finally, osteoprotegerin, a regulator of osteoclastogenesis, appears to be a soluble receptor for TRAIL [18]. The idea of targeting specific death receptors to induce apoptosis in tumours is attractive; thus it is particularly intriguing to explore how a complex family of death and decoy receptors modulates TRAIL function. Although DR4 and DR5 transcripts and TRAIL mRNA are expressed in many tissues, most normal cells are resistant to apoptosis induction by this ligand [9,10]. Therefore DcR1 and DcR2 receptors may contribute to physiological resistance to TRAIL. In contrast, several tumour cell lines express DR4 and DR5, but little DcR1 and DcR2, suggesting that cancer cells are more sensitive to the TRAIL apoptotic signal. However, some tumour cells can acquire resistance to TRAIL-induced apoptosis by up-regulating decoy receptor expression [19]. Androgens are critically involved in the development, progression, and treatment of prostate cancer [20]. Currently, a major therapy for the treatment of localized and metastatic prostate cancer is androgen ablation, which induces extensive apoptosis of androgen-dependent prostate cancer cells, resulting in tumour regression and improved prognosis [21,22]. Androgen deprivation by castration induces cell death in hormone-sensitive rat ventral prostate [23], and caspase-3 and -6 expression and activation are targeted by hormone action during this process [24]. The involvement of the death receptor pathway in ventral prostate apoptosis has been well studied [25-27]. We reported earlier that testosterone specifically controls DcR2 mRNA and protein expression in normal adult rat ventral prostate, and concluded that androgen withdrawal by reducing DcR2 expression might leave the cells vulnerable to the cell death signal generated by TRAIL via its functional receptors [28].

Although androgen regulation of human TRAIL receptor expression under pathological conditions has not been reported, we can hypothesize on the basis of our previous results that hormonal variations in DcR2 receptor expression levels are involved as inhibitor/inducer in TRAIL-induced apoptosis in prostate cancer cells. We have investigated the role of the decoy receptor DcR2 in apoptosis induction by TRAIL in human prostate cancer, using the androgen-sensitive cell line, LNCaP.

Initially the dose-dependent effects of androgen on TRAIL system expression showed that the hormone sensitivity of DcR2 and DR5 protein expression correlates with TRAIL-induced apoptosis. After silencing DcR2 protein expression with siRNA, we explored the consequences of the downregulation on TRAIL-induced apoptosis in LNCaP cells.

## Results

### Sensitivity of LNCaP Cells to Androgen Concentration

**Figure 1:**
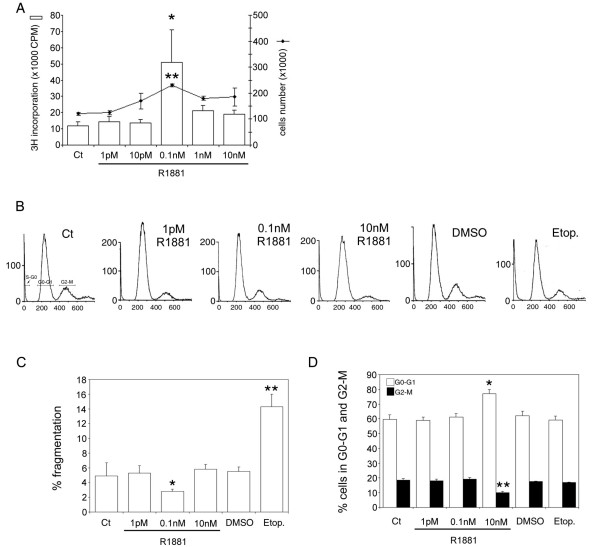
**Androgen control of LNCaP cell proliferation**. (A): cells were treated with increasing concentrations of R1881 (1 pM-10 nM) or ethanol as the control vehicle. Proliferation was measured 4 days later by [3H]-thymidine incorporation with *P < 0.005 versus other concentrations and by cell number counting with **P < 0.05 versus other concentrations. (B): cell cycle was analysed by flowcytometry after treatment with R1881 at 1 pM, 0.1 nM, and 10 nM, with its ethanol vehicle as control (Ct) (0.001% was the highest concentration used), and with 300 μM Etoposide (positive control) and its DMSO diluent at 0.03%. A representative cell cycle profile is presented. (C) and (D): Cell cycle distribution analysis; the values are expressed as the mean ± SD determined from 2 independent experiments, with triplicate cultures per treatment condition. (C): percentage DNA fragmentation (S-G0), *P < 0.005 versus control and R1881 concentrations, **P < 0.005 versus DMSO. (D): percentage of cells in G0-G1 and in G2-M with *P < 0.005 and **P < 0.005 versus the other treatment conditions, respectively.

The effect of androgen on TRAIL system expression in hormone-sensitive prostate cancer cells was examined using human hormone-sensitive LNCaP cells as a model. These cells gave a bell-shaped growth curve in response to increasing doses (1 pM-10 nM) of R1881 (Figure 1A). R1881 concentrations, in the pM range, induce cell proliferation, with a significant maximum at 0.1 nM (^3^H incorporation up 4.25-fold, *P *< 0.002, and cell number increased 1.9-fold compared with controls, *P *< 0.05), whereas doses in nM range impeded cell growth. DNA content was assessed by flowcytometry (Figure 1B). Under the best conditions of cell proliferation (0.1 nM R1881), there was less DNA fragmentation (*P *< 0.005) than at other concentrations (Figure 1C); and globally, compared with control cells, the absence of a significant increase in the DNA fragmentation percentage under all conditions indicated the absence of apoptosis. Incubation in 10 nM R1881 produced the highest level of cells in G0-G1 (+28%, *P *< 0.005) and the lowest level of cells in G2-M (-50%, *P *< 0.005) in comparison with controls (Figure 1D), confirming this concentration as inhibiting cell growth. In the positive control, etoposide, the increase in the DNA fragmentation percentage (Figure 1C) correlated with a decrease in the percentage of cells distributed in the 2 corresponding G0-G1 and G2-M stages (Figure 1D), compared with the DMSO control. Except in the positive control (etoposide), and in accordance with the absence of apoptosis under all other conditions, cleaved caspase-3 protein levels were unchanged (data not shown).

### Androgen Action on TRAIL System Protein Expression in LNCaP Cells

**Figure 2:**
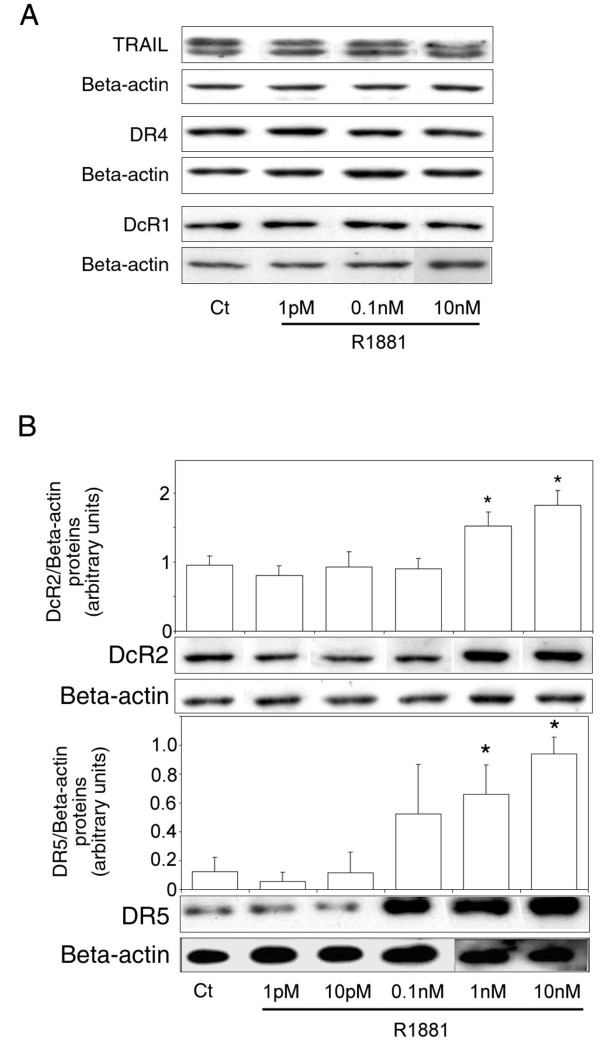
**Androgen control of TRAIL and TRAIL-receptor protein expression in LNCaP cells**. Cells were treated for 4 days with increasing concentrations of R1881 (1 pM-10 nM) or with ethanol vehicle as control (Ct) (0.001% was the highest concentration used). Proteins were characterized by immunoblot analysis using anti-TRAIL, anti-DR4 and anti-DcR1 antibodies (A), and with anti-DcR2 and anti-DR5 antibodies (B). Histograms represent corresponding protein levels and are normalized for β-actin expression. The values represent the mean ± SD determined from triplicate cultures of a representative experiment from 3 independent experiments. For each protein a representative autoradiograph is shown. *P < 0.005 compared with control.

To investigate the effect of androgen on TRAIL, DR4, DR5, DcR1 and DcR2 protein levels, hormone-sensitive LNCaP cells were cultured in increasing concentrations of R1881 (1 pM-10 nM). Ligand and receptors protein levels were measured by Western blot analysis (Figure 2). No significant variations were observed in TRAIL (present as a doublet), DR4 and DcR1 protein levels (Figure 2A), whereas DcR2 and DR5 levels were significantly increase in the nanomolar range compared to the control (1.6-fold, *P *= 0.002, and 5.6-fold, *P *= 0.003, respectively, at 1 nM; Figure 2B). All the samples were run and detected together, but some films were cut and pasted to remove spots from replicate cell protein extracts. A correlation was found between androgen concentration and DcR2 and DR5 protein expression that corroborated our previous results obtained in normal prostate (modulation of DcR2 expression [28]) and testis (modulation of DR4, DR5 and DcR2 expression [29]), another hormone-sensitive tissue.

**Figure 3:**
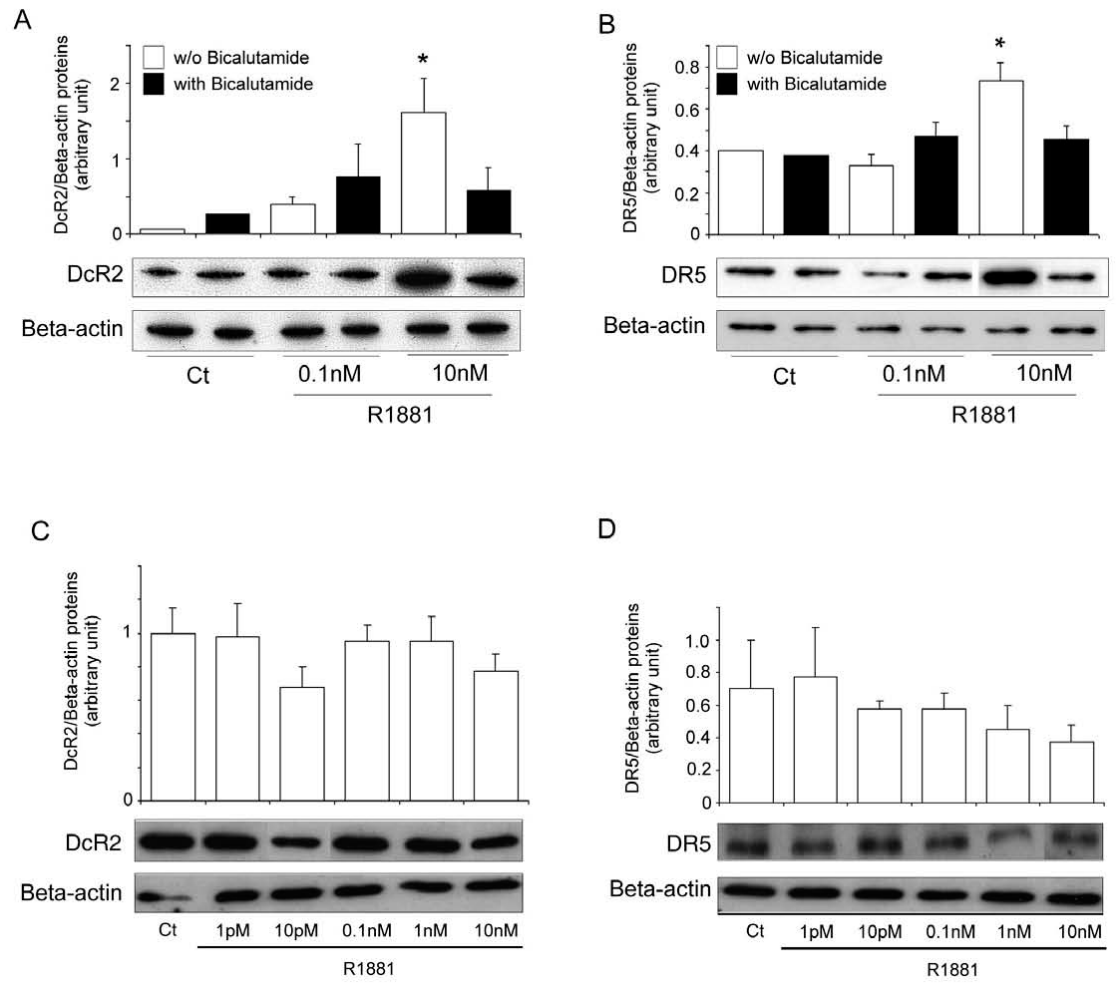
**DcR2 and DR5 protein expression in prostate cancer cells when androgen action had been neutralised**. (A) and (B): in LNCaP cells incubated for 4 days with ethanol vehicle as control (Ct) (0.001% was the highest concentration used) or R1881 at 0.1 nM or 10 nM, without or with 25 μM antiandrogen bicalutamide. (C) and (D): in PC3 cells, devoid of androgen receptor, incubated for 4 days with ethanol vehicle as control or increasing concentrations of R1881. All the samples were run and detected together, but some films were cut and pasted to remove spots from replicate cell protein extracts. Immunoblot results and histograms represent DcR2 and DR5 levels and were normalized to β-actin expression. The values represent the mean ± SD determined from triplicate cultures of a representative experiment from 2 independent experiments. For each protein a representative autoradiograph is shown. *P < 0.05 versus the other treatment conditions.

There are no reports of androgen responsive elements in TRAIL receptor promoters. However, the following experiments show that DcR2 and DR5 expression can be regulated via the androgen receptor. LNCaP cells incubated simultaneously with R1881 and the specific androgen receptor antagonist, bicalutamide (25 μM), significantly abrogated the increase in DcR2 and DR5 protein expression due to 10 nM R1881 alone (Figure 3A and 3B). Similarly, the DcR2 and DR5 expression after R1881 incubation was not significantly modified in prostate cancer cells devoid of androgen receptors, such as PC3 cells (Figure 3C and 3D).

### Androgen Regulates Apoptosis Induction by TRAIL in LNCaP cells

**Figure 4:**
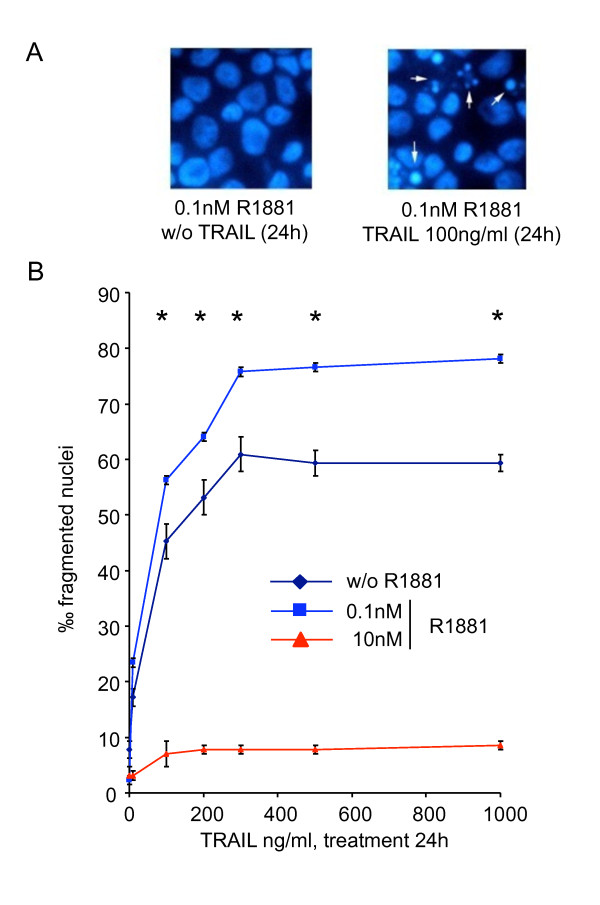
**Androgen regulation of TRAIL-induced apoptosis in LNCaP cells**. After 4 days of culture with ethanol vehicle (Ct: control), or R1881, LNCaP cells were treated with increasing TRAIL concentrations for 24 h. (A): fragmented nuclei were investigated after DAPI staining by fluorescence microscopy. (B): the number of fragmented nuclei was determined in LNCaP treated with increasing TRAIL concentrations for 24 h. The values represent the mean ± SD of fragmented nuclei per 1,000 cells after analysis of 5,000 randomly selected cells per culture and determined from 5 replicate cultures of a representative experiment from 3 independent experiments. *P < 0.005 versus TRAIL treated cells in androgen free and 10 nM R1881 conditions.

To investigate TRAIL-mediated cell death related to androgen concentration, LNCaP cells were cultured for 4 days in 0.1 nM or 10 nM R1881 before being treated with increasing TRAIL ligand concentrations from 10 ng to 1 μg for 24 and 48 h. Cell death was monitored by DAPI staining (Figure 4A). Androgen at 0.1 and 10 nM were chosen because they represented the best and the poorest cell growth conditions, respectively (Figure 1), and because of the variations in DcR2 and DR5 protein levels (Figure 2). In comparison with androgen-free conditions (Figure 4B), the percentage of fragmented nuclei in the presence of 0.1 nM R1881 increased significantly between 10 and 200 ng TRAIL per milliter (+28% and + 38% for TRAIL 100 ng and 200 ng, respectively; *P *< 0.005) to reach a plateau at 300-1000 ng/ml. TRAIL-induced apoptosis was not assessed at 10 nM R1881. Results obtained after 48 h treatment were slightly higher than after 24 h (data not shown). Consequently, the best TRAIL-induced apoptosis conditions for LNCaP cells were 100 ng of TRAIL over 24 h.

### DcR2 Protein Expression is Correlated with TRAIL-Sensitivity in Prostate Cancer LNCaP Cells

**Figure 5:**
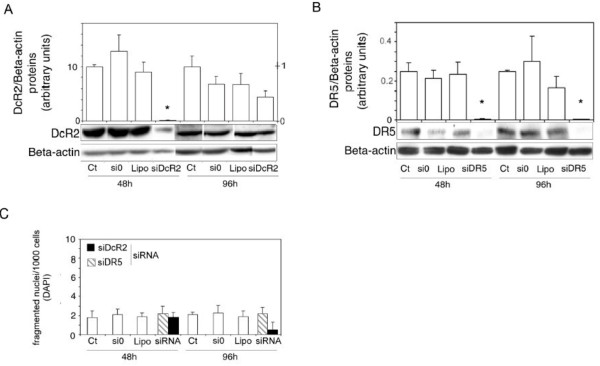
**SiRNAs specific for DcR2 and DR5 suppress gene expression in LNCaP cells**. Gene-specific siRNAs and scrambled siRNAs controls (si0) (0.5 μg) were added to the media using lipophilic transfection-enhancing reagent (lipofectamine/lipo) for 4 h, and then the cells were cultured in media containing 0.1 nM R1881. Cells were harvested after 48 or 96 h for immunoblot analyses with DcR2 (A) and DR5 (B) specific antibodies. The blots were reprobed with antibody against ß-actin to confirm equal protein loading (15 μg). All the samples were run and detected together but 96 h films were cut and pasted to remove spots from replicate cell protein extracts. Representative autoradiograph results are shown and histograms represent DcR2 (right scale: 48 h incubation; left scale: 96 h incubation) and DR5 protein levels and were normalized for ß-actin expression. (C): the number of fragmented nuclei per 1,000 cells was determined in all transfection treatment conditions (scrambled and specific siRNAs and lipofectamine) with DAPI method. The values represent the mean ± SD determined from triplicate cultures of a representative experiment from 3 independent experiments. *P < 0.005 versus the other treatment conditions.

We have reported a correlation between androgen status, apoptosis and DcR2 expression in rat prostate [28], and postulated that DcR2 decoy receptor expression may be directly related to TRAIL-induced apoptosis resistance in prostate cancer cells. If so, the downregulation of DcR2 protein expression could reverse resistance to TRAIL-induced cell death. Since DcR2 and DR5 protein expression in LNCaP cells is androgen sensitive (Figure 2), we tested the previous hypothesis by suppressing DcR2 gene expression using siRNA (Figure 5). Similarly, the role of DR5 was investigated to check its function and the efficiency of the silencing method. DcR2 (Figure 5A) and DR5 (Figure 5B) proteins were knocked down after transfection with the corresponding siRNAs, which were followed by 48 h incubation in 0.1 nM R1881 (-98% and -92%, respectively, in comparison with controls (untreated and si0 treated cells), *P*< 0.005). Protein silencing was less efficient after 96 h incubation than at 48 h because of the decrease in siRNA strands and exhaustion of the medium. No significant variations were observed in fragmented nuclei per thousand DAPI-stained cells treated with siDcR2 or siDR5 alone, or with the different transfection reagents (scrambled siRNAs/si0 and specific siRNAs, lipofectamine) (Figure 5C), indicating that there were not toxic materials present.

**Figure 6:**
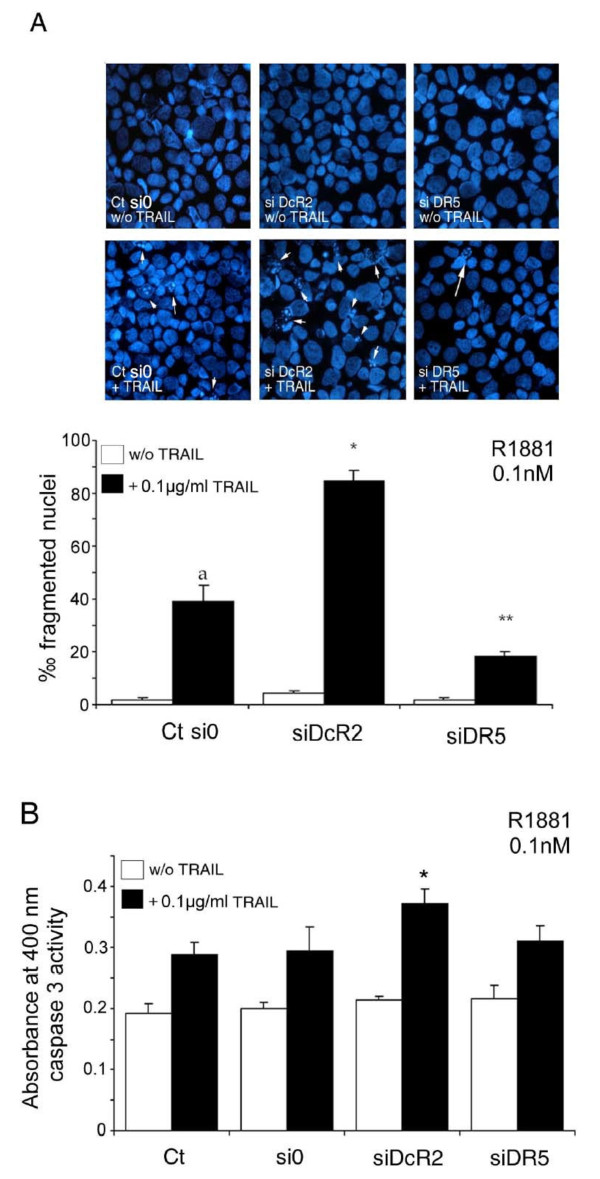
**Inhibition of DcR2 and DR5 protein expression by siRNA sensitizes LNCaP cells to TRAIL-induced apoptosis in medium containing 0.1 nM R1881**. LNCaP cells, untransfected (Ct: control) or transfected with scrambled siRNA (si0) or siDcR2 or siDR5 (0.5 μg) for 48 h were treated with TRAIL (100 ng/ml) for 24 h (A). White arrows show fragmented nuclei detected by the DAPI method. Histograms represent the number of fragmented nuclei per 1,000 cells from 5,000 randomly selected cells per culture. The values represent the mean ± SD determined from triplicate cultures of a representative experiment from 3 independent experiments. a, *P *< 0.005, *P < 0.005 and **P < 0.005 versus the other treatment conditions. TRAIL-induced apoptosis, under previous conditions, was controlled by caspase-3 activity assay (B). Histogram values represent the mean ± SD determined from triplicate cultures of a representative experiment from 2 independent experiments performed at 0.1 nM R1881, *P < 0.005 versus the other treatment conditions.

The 48 h silencing of the DcR2 Protein as described in Figure 5, followed by treatment with TRAIL as described in Figure 4, led to a significant increase in fragmented nuclei (2.13-fold, *P *< 0.002) in transfected LNCaP cells in comparison with untransfected control cells or scrambled siRNA transfected cells (Figure 6A). This was confirmed by a significant increase in caspase 3 activity in DcR2 siRNA transfected cells and treated with TRAIL (+26%, *P *< 0.005) in comparison with scrambled siRNA transfected or untransfected control cells with TRAIL, (Figure 6B). Silencing of the DR5 protein resulted as anticipated in a significant reduction in TRAIL-induced apoptosis in comparison with untransfected control cells and scrambled siRNA transfected cells (-54%, *P *< 0.002), and no significant variation in caspase 3 activity (Figure 6A and 6B, respectively).

**Figure 7:**
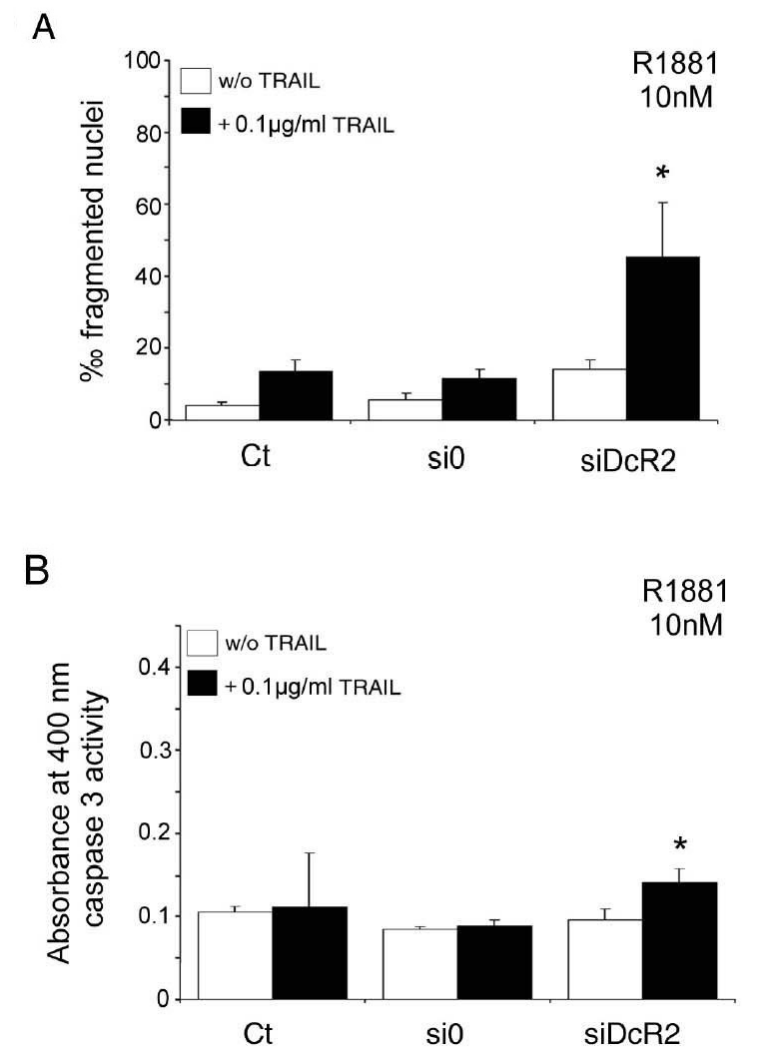
**Inhibition of DcR2 protein expression by siRNA sensitizes LNCaP cells to TRAIL-induced apoptosis, in medium containing 10 nM R1881**. LNCaP cells, untransfected (Ct: control) or transfected with scrambled siRNA (si0) or siDcR2 (0.5 μg) for 48 h were treated with TRAIL (100 ng/ml) for 24 h. (A) Histograms represent the number of fragmented nuclei per 1,000 cells from 5,000 randomly selected cells per culture. The values represent the mean ± SD determined from triplicate cultures of a representative experiment from 3 independent experiments. *P < 0.005 versus the other treatment conditions. (B) TRAIL-induced apoptosis, under previous conditions, was controlled by caspase-3 activity assay. Histogram values represent the mean ± SD determined from triplicate cultures of a representative experiment from 2 independent experiments performed at 10 nM R1881, *P < 0.05 with the Fisher post-test versus the other treatment conditions, except the control with TRAIL.

LNCaP cells cultured in 10 nM R1881 showed no apoptosis without (Figure 1C) or with TRAIL peptide (Figure 4B), and an increase in DcR2 protein levels (Figure 2B). Silencing of DcR2 protein at 10 nM R1881 resulted in partial recovery of TRAIL-induced apoptosis in comparison with untransfected control cells and with scrambled siRNA transfected cells (3.1-fold and 3.8-fold, respectively, *P *< 0.005; Figure 7A), accompanied by a significant increase in caspase 3 activity in comparison with si0 transfected cells (+59%, *P *< 0.05; Figure 7B). This suggests that DcR2 upregulation rendered LNCaP cells more resistant to TRAIL-mediated apoptosis, whereas downregulation of DcR2 protein enhanced TRAIL-induced apoptosis.

## Discussion

In the pathological prostate, the physiological regulation of apoptosis by TRAIL in relation to death and decoy receptor expression and hormonal concentration is unknown. Our previous results [28] showed that androgen deprivation associated with an apoptotic process led to a decrease in DcR2 expression in normal adult rat hormone sensitive ventral prostate. The downregulation of DcR2 expression following androgen deprivation was thought to leave androgen sensitive cells vulnerable to the cell death signal induced by TRAIL via its pro-apoptotic receptors.

In the presence of R1881 at 1 pM-10 nM, the growth curve LNCaP cells was characteristically bell-shaped, with a peak at 0.1 nM [30,31]. TRAIL, DR4 and DcR1 protein expressions were unaffected under these conditions, whereas DR5 and DcR2 protein expression increased over a nanomolar range. No androgen responsive elements have been reported in TRAIL-receptor promoters, but several experimental results suggest the genomic regulation of DR5 and DcR2 expression by androgens occurs in the same way as their inhibition by the anti-androgen, bicalutamid, and receptor expression remains unmodified in PC3 cells incubated with androgen. However, its regulation by an (as yet unidentified) androgen-dependent transcription factor is possible, and the increased DcR2 protein level in LNCaP cells may be due to its regulation by an (also as yet unidentified) androgen-dependent translational factor.

TRAIL-induced apoptosis of LNCaP cells is maximal at 0.1 nM R1881. Increases in DR5 and DcR2 receptor expression at R1881 concentrations of up to 0.1 nM may be related to the absence of TRAIL induced apoptosis in situations of poor cell proliferation. Silencing of hormone sensitive receptors with siRNA shows that, at 0.1 nM R1881, and when DcR2 protein expression is silenced by the corresponding siRNA, TRAIL-induced apoptosis is increased by a factor of 2 in comparison with the controls. In contrast and as expected, apoptosis could be reduced to half when DR5 is silenced, which may be due to pro-apoptotic receptor DR4 expression not being downregulated. DcR2 is therefore a regulator of TRAIL induced apoptosis in the best hormonal concentration for cell growth. Moreover, preliminary results show that the silencing of the DcR2 receptor results in a partial restoration of TRAIL induced apoptosis in otherwise resistant LNCaP cells cultured in 10 nM R1881. A partial restoration of TRAIL mediated apoptosis in 10 nM androgen may be due to poor cell growth involving an intracellular signalling and trafficking that is much slower than in the favourable growth conditions (0.1 nM) in vitro.

Rokhlin et al. [32] showed that the expression level of the DR4 and DR5 receptors in LNCaP incubated with fetal calf serum or 100 nM dihydrotestosterone was clearly superior to the control conditions without androgen, whereas the decoy receptor expression level was not given. Although these authors [33], like others [34], found resistance of LNCaP cells to TRAIL-induced apoptosis, interestingly they also showed that FADD and caspase 8 recruitment for TRAIL-DISC formation and cell death induced by the TRAIL ligand were androgen-related in LNCaP cells [32,35]. These results along with ours support the hypothesis that LNCaP sensitivity to the TRAIL ligand is androgen-regulated, and related to the expression of its receptors (decoy and pro-apoptotic) and DISC formation [32,35]. In contrast, no significant variations for DR4 expression were observed under our conditions with R1881 present under 10 nM, which may be due to differences in experimental conditions (androgen concentration, pH, media composition, temperature, timing of treatment, presence of PI3K/Akt pathway inhibitor, etc.). The relative contribution of each death receptor to apoptosis induction in cells expressing both receptors remains unknown [4]. *In vitro*, comparison of TRAIL-death receptors cell surface expression and TRAIL sensitivity of cancer cell lines did not show any consistent pattern, suggesting that TRAIL sensitivity may be dependent on a permissive environment for preferential signalling via TRAIL-DR4 or DR5 receptors expression controlled by other intracellular mechanisms in resistant cells [36,37]. Our data indicates that androgens play a role in apoptosis sensitivity in LNCaP cells, and their resistance to TRAIL-induced apoptosis is more closely linked to the upregulation of the decoy receptor DcR2 expression than to downregulation of pro-apoptotic receptors.

Considering the context of the pathological and hormone-sensitive prostate, these results, like others [38], show that the resistance of such tissues to apoptosis is probably more closely linked to increased expression of apoptosis inhibitors than decreased expression of pro-apoptotic proteins. This hypothesis is strengthened by other data showing that the silencing of Bcl2, FLIP or IAPs (XIAP and survivine) by siRNAs sensitizes resistant melanoma cells to TRAIL-induced apoptosis [39]. In the same way, Sung et al. [40] reported that celastrol, a triterpene, enhanced TRAIL-induced apoptosis through the downregulation of cell survival proteins and upregulation of death receptors. Hesry et al. [41] showed that sensitivity to the TRAIL ligand is related to tumour progression (in LNCaP, DU 145, PC3 cells) and that TRAIL-induced cell death is only linked to the DR5 receptor, moreover they found DcR1 receptor expression to be undetectable, whereas DcR2 was significantly more abundant in tumour cells than non-neoplastic cells, and may thus contribute to the partial resistance to TRAIL found in some prostate tumour cells (e.g. LNCaP and DU 145). Interestingly, another study [42] reported that the combined treatment of prostate cancer cells (LNCaP and PC3) with TRAIL and chemotherapeutic agents overcame their resistance by triggering caspase activation; in the same way Sanlioglu et al. [43] indicated that DcR2 siRNA and adenovirus delivery of TRAIL dramatically affected the tumorigenic potential of prostate cancer LNCaP and DU 145 cells.

If androgen withdrawal in prostate cancer treatment decreases anti-apoptotic gene expression as DcR2 in androgen-dependent cells, this could induce apoptosis, resulting in prostate tumour reduction. Over the middle term and following androgen withdrawal [44], it is conceivable that androgen-independent cancer cells might arise as a subpopulation that has acquired the capacity to upregulate the expression of decoy receptors and perhaps other anti-apoptotic proteins, such as XIAP [45]; in this way they might escape anti-tumour surveillance by immune cells. Under these conditions, upregulation of anti-apoptotic proteins would be independent of androgen deprivation. Furthermore, the identification of drugs or experimental methods (downregulation by siRNAs) that decrease TRAIL decoy receptor expression for a long time after androgen ablation may prove to be useful therapeutically on their own, or in combination with other approaches (molecular/protein targets, cytotoxic agents, etc.) to induce apoptosis as treatment for prostate cancer and/or for otherwise resistant cancer cells.

## Conclusions

The differential sensitivity of tumour cells to TRAIL-induced apoptosis may be mediated by the modulation of surface TRAIL receptor expression that could be related to the androgen concentration in prostate. Decoy (DcR2) and death (DR5) receptor protein expression is correlated with hormone concentrations and TRAIL-induced apoptosis in LNCaP cells. Silencing of androgen-sensitive DcR2 protein expression by siRNA leads to a significant increase in TRAIL-mediated apoptosis related to androgen concentration in LNCaP cells. The data support the view that hormone modulation of DcR2 expression is involved in regulating TRAIL-induced apoptosis in the androgen-sensitive prostate carcinoma cell line, LNCaP, and provides preliminary insight into cell death induction in apoptosis resistant hormone-sensitive prostate cancer cells. TRAIL action and DcR2 expression modulation are potential of clinical value for advanced tumour treatment when androgen-deprivation therapy fails, leading to recurrent tumour growth in a hormone-refractory manner.

Furthermore, the identification of drugs or experimental methods that enhance TRAIL-induced apoptosis through the downregulation of TRAIL decoy receptor or cell survival proteins expression may prove themselves to be therapeutically useful, or in combination with other therapeutic approaches to induce apoptosis as treatment for prostate cancer or for otherwise resistant cancer cells.

## Materials and methods

### Cell Culture

Human prostatic carcinoma cell lines, LNCaP and PC3, were obtained from the American Type Culture Collection (ATCC, Bethesda, MD). Cells were maintained in RPMI 1640 medium (Invitrogen, Cergy-Pontoise, France), supplemented with 7.5% fetal calf serum (FCS; Invitrogen), 2 mM L-Glutamine (Sigma, Isle d'Abeau, France), 20 μg/ml streptomycin, 20 U/ml penicillin (Invitrogen), and 50 U/ml Nystatin (Sigma). LNCaP and PC3 cells were used between passages 50-60 and 18-24, respectively. 17β-hydroxy-17-methyl-estra-4,9,11-trien-3-olone (R1881/testosterone analogue) was purchased from New England Nuclear (Boston, MA) and dissolved in ethanol. Bicalutamide (Casodex, [(2RS)-4'-cyano-3-(4-fluorophenylsulphonyl)-2-hydroxy-2-methyl-3'-trifluoromethyl propioanilide] was a gift from AstraZeneca (Alderly House, Cheshire, England); it was dissolved in 0.2% methanol.

### Cell numeration, proliferation assay and treatment

LNCaP cells (5 × 10^4^) were plated in triplicate per well in 24-well plates (Corning Incorporated, Corning, NY) in steroid-depleted media containing 2.5% charcoal-stripped FCS (HyClone, Logan, UT) for numeration. Cells of each well were counted in duplicate with a Coulter Z1 counter (Beckman Coulter, CA). For [^3^H]-thymidine incorporation, cells were plated in 12-well plates at 12 × 10^4 ^cells/well and incubated with 0.5 μCi/ml [^3^H]-thymidine (Du Pont-New England Nuclear, Les Ulis, France) in medium for 4 h before harvesting. Cells were washed twice with ice-cold PBS and DNA precipitated with 5% trichloracetic acid. The DNA precipitate was dissolved in 0.4 ml of 0.25 M NaOH and incorporated [^3^H]-thymidine was determined by liquid scintillation counting. Sampling at each time-point was in triplicate. For Western-blotting analysis, LNCaP cells were seeded in 10 cm disks (22 × 10^5 ^cells), with 3 disks per condition. Cells were allowed to attach for 24 h and treated with various concentrations of R1881 on day 0. All the cells, including the control cells, were cultured in the presence of the same ethanol concentration (0.001% being the highest concentration used).

### Flowcytometric Analysis

Flowcytometry was used to assess the sub-G1 DNA population of cells undergoing apoptosis. LNCaP cells were seeded at 8 × 10^5 ^cells in 6-cm dishes. After 4 days of culture with or without R1881, cells were collected by trypsinization and fixed in 1% paraformaldehyde for 30 min at 4°C, then in 70% ethanol/30% PBS (phosphate buffer saline) for 2 h at -20°C. Fixed cells were washed with PBS, treated with 30 μg/ml Rnase A in PBS for 60 minutes at 37°C, and propidium iodide in PBS (10 μg/ml) was added. For positive controls, LNCaP were incubated with etoposide (Sigma) diluted with 0.03% DMSO (dimethyl sulfoxide; Sigma). Cell cycle profiles and distributions were determined using a BD Facscan flowcytometer (Becton Dickinson Biosciences, San Jose, CA). Cell cycle distribution was analyzed using Modfit LT software (Verity Software House, Topsham, ME).

### Western-blot analysis

Cell protein extracts were prepared by direct addition of 5 volumes of cold lysis buffer, as previously described [40]. Protein concentration was determined using the Bicinchoninic acid assay reagent (Sigma, Isle d'Abeau, France), with bovine serum albumin as standard. Protein samples (15 μg/well) were resolved by 12% SDS-polyacrylamide gel electrophoresis and electroblotted as described earlier [46]. Nitrocellulose blots were treated and incubated with the following primary antibodies (Santa Cruz Biotechnology, Santa Cruz CA): rabbit polyclonal antibodies raised against human TRAIL or DR4 (1:1,000), goat polyclonal antibodies raised against human DcR1 or DcR2 (1:1,000) and secondary antibodies, horseradish peroxidase (HRP) conjugated goat anti-rabbit IgG (1:2000) or donkey anti-goat IgG (1:5,000) as previously reported [28]. The primary antibody anti-human DR5, obtained from AnaSpec, Incorporated (San Jose, CA), was used at a dilution of 1:1000. Protein loading was checked by reprobing the blot with a rabbit IgG anti-β-actin antibody (1/500) (Sigma). Bound antibodies were detected using the chemiluminescence Western blotting detection kit (Covalab). The Biomax MR films (Eastman Kodak Company, Rochester, NY) were scanned on a Gel doc 2000 apparatus (Biorad, Marnes-la-Coquette, France), and quantified with Quantity one Software Biorad.

### Apoptosis assessment with 4', 6'-diamidino-2 phenylindole staining (DAPI)

LNCaP cells undergoing apoptosis were followed by chromatin condensation, nuclear shrinkage, and formation of apoptotic bodies, as visualized after DAPI staining [47]. Following various specific treatments, the medium was removed and cells fixed in ethanol/acetic acid (3:1, vol/vol) at room temperature for 10 min. Cells were washed with 0.9% NaCl and stained with 0.4 μg/ml DAPI in 0.9% NaCl at room temperature for 30 min and viewed by fluorescence microscopy.

### si RNA

siRNAs (sense and antisense strands) were purchased from Ambion, Inc. (Austin, TX). The sense strand sequences were the following: DR5, 5'-GGACUAUAGCACUCACUGGtt-3'; DcR2, 5'-GGGUGUGGAUUACACCAUUtt-3'; siRNA control was a non-specific random scrambled sequence. *In vitro *transfections used lipofectamine reagent (Invitrogen, Cergy-Pontoise, France). Cells were cultured in 12-well plates, 1 ml/well of 2.5% charcoal-stripped FCS medium. They were allowed to attach for 24 h, the culture medium was then removed and replaced with serum-free RPMI (200 μl). Cells were treated with gene-specific siRNAs and control siRNAs (0.5 μg), plus reagent (2 μl), lipofectamine reagent (1 μl) and 50 μl of RPMI for 4 h at 37°C with 5% CO_2 _in air. Thereafter, 750 μl of 2.5% charcoal-stripped FCS-containing medium were added. Cells were collected or treated 48 h later with TRAIL peptide (PeproTech, Levallois Perret, France). The silencing of targeted proteins was estimated by Western-blot analysis with corresponding antibodies.

### Colorimetric caspase-3 activity assay

The enzymatic activity of caspase-3 was assayed with a Caspase-3 Colorimetric Assay Kit (Alexis Biochemicals, Switzerland). To measure the activity of caspase-3, cell lysates were prepared after their respective treatments. After incubating 200 μg protein of cell lysate per sample in 100 μl of reaction buffer containing DEVD-pNA substrate (200 μM final concentration) at 37°C for 2 h in the dark, absorption was measured at 405 nm.

### Statistical analysis

Statistical analysis were carried out by ANOVA and post-hoc Bonferroni/Dunn tests, except in Figure 3, where significant differences were obtained with the Student-Newman-Keuls post-test (StatView Software; SAS Institute Inc., Cary, NC). Differences were taken as statistically significant at *P *< 0.05. A representative experiment of each series is presented.

Data are expressed as the mean ± SD. Unless otherwise mentioned in the figure legends, the different analyses were performed 3 times with triplicate cultures, and investigated at least 3 times on cells at different passages. TRAIL-induced apoptosis experiments included 5 samples per treatment condition.

## Competing interests

The authors declare that they have no competing interests.

## Authors' contributions

DV performed cell treatments, Western blot analysis, experiments with siRNA, apoptosis assessment with DAPI, statistical analysis and construction of the figures.

MR carried out the caspase-3 activity assay and helped with cell culture. JC and CD performed cell cultures, numeration, proliferation assay and flowcytometric analyses. SY helped to carry out androgen cell treatment. AR and MD participated in the design of the study and discussed the results. MB participated in the design, in coordinating the study and discussed its findings. RG, as corresponding author, designed the protocol, helped to carry out experiments, helped to analyse the data during the study and wrote the manuscript. All authors read and approved the final manuscript.
